# A longitudinal study on the changes in the self‐efficacy of breast cancer patients during adjuvant chemotherapy

**DOI:** 10.1002/nop2.1533

**Published:** 2022-11-30

**Authors:** Chiao‐Chi Kuan, Wen‐Hung Kuo, Shu‐Hui Chang, Huey‐Fang Sun

**Affiliations:** ^1^ Department of Nursing National Taiwan University Cancer Center Taipei City Taiwan; ^2^ Graduate Institute of Medical Sciences, School of Nursing, National Defense Medical Center Taipei City Taiwan; ^3^ Department of Surgery National Taiwan University Hospital Taipei City Taiwan; ^4^ Institute of Epidemiology and Preventive Medicine, College of Public Health National Taiwan University Taipei City Taiwan; ^5^ School of Nursing, National Defense Medical Center Taipei City Taiwan

**Keywords:** adjuvant chemotherapy, breast cancer, generalized linear model, longitudinal study, self‐efficacy

## Abstract

**Aim:**

We aimed to investigate the changes in the self‐efficacy of patients with breast cancer (BC) during adjuvant chemotherapy.

**Design:**

A longitudinal study was conducted.

**Methods:**

One hundred thirty patients with BC who underwent postoperative adjuvant chemotherapy at a medical centre in northern Taiwan were evaluated using self‐efficacy survey tools before the first (T1), second (T2) and last (T3) cycles of adjuvant chemotherapy. The independent t‐test, Kruskal–Wallis test, one‐way analysis of variance, Pearson's correlation coefficient and generalized estimating equation were used for data analysis.

**Results:**

The self‐efficacy measures of patients with BC were significantly higher at both T2 and T3 than at T1 (*p* < 0.001). Religious beliefs and changes in self‐efficacy showed a significant correlation (*p* = 0.04). These findings will facilitate timely interventional measures to improve self‐efficacy in patients with maladaptive behaviours, alleviate psychological distress and reduce the risk of future disease recurrence.

## 1 INTRODUCTION

According to the Global Cancer Observatory 2018 estimates of cancer incidence and mortality produced by the International Agency for Research on Cancer, breast cancer (BC) is the most common cancer among women in 154 countries (Bray et al., 2018). BC poses a major threat to women's health and requires active prevention and intervention by health institutions and medical personnel. In Taiwan, the incidence of BC has been the highest among women with cancer since 2003. The age of the patients at the time of their BC diagnosis in Taiwan had a range of 45–69 years (median = 53 years) and was approximately 10 years earlier than that in Western countries (Health Promotion Administration, 2020).

## 2 BACKGROUND

In most patients with BC, postoperative adjuvant chemotherapy is an important treatment strategy. The literature recommends that patients with BC should undergo adjuvant chemotherapy within 3–6 weeks after surgery based on their tumour genotype and prognostic factors, which in turn depend on the tumour burden and tumour biology (Cardoso et al., 2019). Adjuvant chemotherapy aims to destroy residual cancer cells that are invisible to the naked eye, reduce the rate of future recurrence and increase the survival rate (Early Breast Cancer Trialists' Collaborative Group, 2005; NCCN, 2020).

The side effects of chemotherapy are a source of intolerable stress and fear for most patients with BC (Kim et al., 2018; Petrelli et al., 2012), especially those undergoing adjuvant chemotherapy for the first time. These patients often experience feelings of helplessness and anxiety due to a lack of security and confidence, which not only affects their quality of life but may also hinder the progress of planned medical care (Papadopoulou et al., 2017; Singer et al., 2015). A questionnaire‐based follow‐up study on the fear of treatment (including surgery, chemotherapy and radiotherapy) among 761 German patients with primary BC showed that they feared chemotherapy more than surgery or radiotherapy and that this state of elevated fear persisted until chemotherapy was completed (Singer et al., 2015).

The self‐management ability of patients with cancer plays a major role in the implementation of cancer treatment plans (Foster et al., 2015). Individual self‐management behaviours are closely associated with self‐efficacy (Yu et al., 2014), that is the belief that one can perform a certain task. Bandura proposed that self‐efficacy is an effective predictor of behaviour that reflects how an individual thinks, feels and is motivated to perform actions (Bandura, 1977). When faced with difficulties, the continuity and likelihood of success of healthy behaviours are higher in individuals with high self‐efficacy than in those with low self‐efficacy. Self‐efficacy can promote empowerment and motivate individuals to effectively perform specific actions, thus allowing them to achieve their desired results and improve their quality of life (Bandura, 1977; O'Leary, 1985).

Previous studies have reported that self‐efficacy is associated with physical and mental abilities to adapt and plays a major role in the behaviour of individuals. Self‐efficacy is closely associated with the daily self‐management ability of an individual to adapt to and manage stressful life events (Huang et al., 2018). Studies have shown that higher levels of self‐efficacy are associated with improved self‐management and disease adaptation abilities (Peters et al., 2019), more effective improvement of life satisfaction and low levels of mental distress (Curtis et al., 2014). In clinical practice, strategies for self‐efficacy have been utilized to improve self‐management ability as well as to enhance treatment and disease prevention (Foster et al., 2015) in many non‐BC patients, such as those with prostate cancer (Curtis et al., 2014), diabetes (Al‐Hashmi et al., 2018) and chronic diseases (Jerant et al., 2008). With respect to the response to life events and stress‐inducing situations, individuals must maintain a positive attitude when faced with stressful life events and continue to create successful life experiences to improve their own self‐efficacy (Brandão et al., 2017), face the undesirable effects of the disease (Papadopoulou et al., 2017), increase treatment compliance (Peters et al., 2019) and maintain good quality of life (Rottmann et al., 2010). Conversely, individuals who avoid life problems and have negative attitudes often achieve negative results and trigger anxiety, depression and physical and mental distress.

A cross‐sectional survey of 97 newly diagnosed patients with BC who underwent a mastectomy was conducted during their chemotherapy using the general self‐efficacy scale (GSES, which ranges from 10 to 40), which showed an average score of 27.15 and revealed a positive correlation between self‐efficacy and self‐management behaviours (Zhang & Schwarzer, 1995). It has also been demonstrated that self‐efficacy was positively correlated with self‐care behaviour and that self‐efficacy facilitates the achievement of self‐care goals and adaptation to illness while controlling for the effects of uncertainty (Zhang et al., 2015). Self‐efficacy effectively alters and enhances the lifestyles of patients with BC, enabling them to manage their physical and psychological challenges and the undesirable effects of chemotherapy and to coexist with cancer (Zhang et al., 2015). Another cross‐sectional study on the correlation between self‐efficacy and fear of disease recurrence in 118 newly diagnosed patients with BC in Germany (stages I–IV) found that self‐efficacy was negatively correlated with a fear of disease progression (Melchior et al., 2013).

High self‐efficacy can improve the quality of life of cancer patients during chemotherapy, reduce anxiety and depression and promote pleasure (Papadopoulou et al., 2017). However, longitudinal studies on the degree of self‐efficacy in patients with BC during adjuvant chemotherapy are limited. Therefore, we aimed to address this research gap by adopting a longitudinal study design to track the changes in self‐efficacy of patients with BC at three time points, starting from the point prior to postoperative adjuvant chemotherapy and concluding after the treatment course. This study aimed to understand the status of self‐efficacy of patients with BC during adjuvant chemotherapy, provide empirical evidence as a reference for clinicians and promote the development of self‐efficacy intervention measures for patients with BC, thereby effectively reducing the physical and mental stress experienced by patients and improving their quality of life.

## 3 METHODS

### 3.1 Study design and participants

A longitudinal study design was used. Female patients with newly diagnosed BC were referred to the study by physicians at the breast surgery clinic of a medical centre in northern Taiwan. The inclusion criteria were as follows: (1) stage I to stage III BC diagnosed by specialists; (2) undergoing a course of adjuvant chemotherapy for the first time; (3) the chemotherapy agents used were anthracyclines or taxanes; and (4) patients were conscious females who were able to speak in Mandarin or Taiwanese and were ≥20 years of age. We excluded patients with a history of mental illness. A total of 134 patients were included over the research period, of which 130 completed the study.

We used purposive sampling and structured questionnaire interviews, and each interview and questionnaire evaluation took approximately 30–40 min.

### 3.2 Sample size calculation

This study used G*Power software (ver. 3.1; Heinrich‐Heine‐Universität Düsseldorf, Düsseldorf, Germany) for the sample size estimation. According to previously proposed standards, the sample size that was calculated based on an effect size of 0.2 (medium–low effect size values), power of 0.8, and 13 predictors (Cohen 1977), required 98 individuals. The expected sample size loss to follow‐up rate of 30% was applied; therefore, the final sample size was 130 individuals.

### 3.3 Main research tools

#### 3.3.1 Basic characteristics of research participants

We collected data for the basis characteristics of the participants, including their age, educational attainment, marital status, employment status, religious beliefs, income, support group participation, type of chemotherapeutic agent, disease stage, BC subtype and other chronic diseases.

#### 3.3.2 General self‐efficacy scale

The Chinese version of the GSES was used as a study tool. The scale included 10 items for overall assessment of an individual's confidence in the implementation of future actions (Zhang & Schwarzer, 1995). A 4‐point Likert scale was used, with 1 indicating “completely disagree,” 2 indicating “somewhat agree,” 3 indicating “mostly agree” and 4 indicating “completely agree.” The total score ranged between 10 and 40, with lower scores indicating lower self‐efficacy and higher scores indicating higher self‐efficacy. The different language versions of the GSES exhibit unidimensionality; thus, they contain only one general factor, and a single factor can account for >50% of the total variance. The Chinese version of the GSES has demonstrated Cronbach's *α* of 0.91 in previous research (Schwarzer et al., 1997). The explanatory power of the overall scale in this study was 78.03%, and Cronbach's *α* at the three measurement time points ranged from 0.94 to 0.96. In this study, Cronbach's *α* was 0.97. The scores of the items on the self‐efficacy scale are shown in Table 1.

**TABLE 1:** Items of self‐efficacy and Cronbach's alpha

Item of self‐efficacy	Cronbach's alpha (if item is deleted)
1. I can always manage to solve difficult problems if I try hard enough.	0.966
2. If someone opposes me, I can find the means and ways to get what I want.	0.965
3. It is easy for me to stick to my aims and accomplish my goals.	0.964
4. I am confident that I could deal efficiently with unexpected events.	0.963
5. Thanks to my resourcefulness, I know how to handle unforeseen situations.	0.964
6. I can solve most problems if I invest the necessary effort.	0.964
7. I can remain calm when facing difficulties because I can rely on my coping abilities.	0.964
8. When I am confronted with a problem, I can usually find several solutions.	0.963
9. If I am in trouble, I can usually think of a solution.	0.963
10. I can usually handle whatever comes my way.	0.964

### 3.4 Data collection and ethics

This study was approved by the Institutional Review of the [REDACTED] Hospital (No. [REDACTED]) for data collection between August 2017 and September 2018. Patients who met the inclusion criteria were referred to the researchers by their attending physicians who specialized in breast health, who explained the content of the research protocol to the patients. The researchers obtained verbal consent from the patients to participate in the study and received signed letters of informed consent from all patients. During the interview, the researchers collected information on the basic characteristics of the patient (including age, employment status, educational attainment, economic status, religious beliefs, treatment type, type of chemotherapeutic agents, number of chemotherapy cycles and support group participation) as well as whether the patient was undergoing adjuvant chemotherapy for the first (T1), second (T2) or final time (T3). The GSES was administered in the consultation room of the outpatient clinic.

### 3.5 Statistical analysis

IBM SPSS (ver. 24.0) was used for statistical data analyses. Descriptive statistics included frequency, percentage, mean, standard deviation and boxplots. Inferential statistics included Pearson's correlation coefficient, independent *t*‐test, Kruskal–Wallis test and one‐way analysis of variance. A generalized estimating equation (GEE) was used to analyse whether changes in self‐efficacy at different measurement time points differed significantly.

## 4 RESULTS

### 4.1 Demographic characteristics

The average age of the patients was 52.9 years (standard deviation, 10.38 years; range, 30–78 years). Approximately 31.5% of the patients had university‐level education, and approximately 75.4% were married. Approximately 54.6% of the patients were unemployed, and 71.5% of patients had religious beliefs. Patients with income levels between NT$20000 and 50,000 accounted for 38.5% of the total, followed by those with incomes between NT$60000 and 100,000, who accounted for 27.7% (Table 2).

**TABLE 2:** Demographic characteristics of breast cancer (BC) patients participating in the study (*n* = 130)

Variable	*n*	% (SD)
Age	130	Mean 52.9 (10.38)
Educational attainment		
Middle school or below	25	19.2
High school/vocational school	29	22.3
Junior college	15	11.5
University	41	31.5
Graduate degree or above	20	15.4
Marital status		
Married	98	75.4
Unmarried	22	16.9
Divorced/separated/widowed	10	7.7
Employment status		
Employed	59	45.4
Unemployed	71	54.6
Religious belief		
Yes	93	71.5
No	37	28.5
Income		
<NT$20K	25	19.2
NT$20K–NT$50K	50	38.5
NT$60K–NT$100K	36	27.7
>NT$100K	19	14.6
Support group participation		
Yes	1	0.8
No	129	99.2
Type of chemotherapeutic agent		
Anthracyclines	23	17.7
Taxanes	26	20.0
Anthracyclines and taxanes	81	62.3
Disease stage		
Stage I	51	39.2
Stage II	47	36.2
Stage III	32	24.6
Other chronic diseases		
No	96	73.8
Yes	34	26.2
BC subtype		
Luminal B1	53	40.8
Luminal B2	18	13.8
Triple‐negative	32	24.6
HER2 overexpression	27	20.8

Abbreviations: HER2, human epidermal growth factor receptor 2; SD, standard deviation.

Most of the patients (99.2%) had no previous experience participating in support groups. Approximately 17.7% of the patients were treated with anthracyclines, 20.0% were treated with taxanes, and 62.3% were treated with anthracyclines and taxanes. Stage I BC accounted for 39.2% of all cases, and 36.2% of patients were in stage II. The luminal B1 BC subtype accounted for 40.8% of all BC cases. Approximately 26.2% patients had other concomitant chronic diseases (Table 2).

### 4.2 Relationship between demographic characteristics and self‐efficacy

The results of the correlation analysis between self‐efficacy and the demographic variables at the three time points are shown in Table 3. Except for the significant correlation between religious beliefs and self‐efficacy at T1 (*p* < 0.05), no other variables exhibited a significant relationship. The mean self‐efficacy score of individuals with religious beliefs (2.84) was higher than that of those without religious beliefs (2.50).

**TABLE 3:** Relationships between demographic variables and self‐efficacy

Variable	Self‐efficacy								
	Before T1			Before T2			Before T3		
	*n*	Correlation coefficient	*p* value	*n*	Correlation coefficient	*p* value	*n*	Correlation coefficient	*p* value
Age (a)	130	0.087	0.325	107	0.056	0.565	73	0.102	0.390
		Mean (SD)			Mean (SD)			Mean (SD)	
Educational attainment (d), (c)			0.237			0.994			0.703
Middle school or below (1)	25	2.68 (1.01)		22	3.10 (0.71)		18	3.22 (0.84)	
High school/vocational school (2)	29	2.47 (0.82)		24	3.05 (0.84)		15	3.09 (0.68)	
Junior college (3)	15	2.85 (0.91)		10	3.02 (0.75)		9	3.37 (0.32)	
University (4)	41	2.94 (0.79)		31	3.12 (0.72)		21	3.12 (0.63)	
Graduate degree or above (5)	20	2.77 (0.79)		20	3.08 (0.63)		10	2.92 (0.82)	
Marital status (b)			0.852			0.807			0.205
Married	98	2.74 (0.86)		82	3.08 (0.69)		55	3.20 (0.64)	
Other	32	2.77 (0.90)		25	3.12 (0.82)		18	2.96 (0.81)	
Employment status (b)			0.691			0.772			0.734
Not employed	71	2.72 (0.93)		59	3.10 (0.69)		44	3.16 (0.72)	
Employed	59	2.78 (0.78)		48	3.06 (0.76)		29	3.11 (0.65)	
Religious belief (b)			0.041			0.645			0.460
No	37	2.50 (0.96)		32	3.13 (0.71)		23	3.05 (0.77)	
Yes	93	2.84 (0.81)		75	3.06 (0.73)		50	3.18 (0.66)	
Income (d), (c)			0.061			0.109			0.194
<NT$20 K (1)	25	2.63 (0.96)		22	2.97 (0.73)		20	3.14 (0.87)	
NT$20K–NT$50K (2)	50	2.58 (0.88)		44	3.01 (0.79)		33	3.23 (0.58)	
NT$60K–NT$100K (3)	36	2.83 (0.75)		27	3.08 (0.61)		14	2.82 (0.61)	
>NT$100K (4)	19	3.17 (0.78)		14	3.51 (0.54)		6	3.38 (0.70)	
Type of chemotherapeutic agent (d)			0.152			0.323			0.899
Anthracyclines (1)	23	2.63 (0.84)		19	3.18 (0.76)		15	3.14 (0.56)	
Taxanes (2)	26	3.04 (0.88)		25	3.23 (0.75)		15	3.21 (0.79)	
Anthracyclines and taxanes (3)	81	2.69 (0.85)		63	3.00 (0.69)		43	3.12 (0.71)	
Disease stage (d)			0.741			0.423			0.744
Stage I (1)	51	2.78 (0.86)		43	2.99 (0.73)		27	3.06 (0.69)	
Stage II (2)	47	2.78 (0.87)		36	3.10 (0.64)		26	3.17 (0.69)	
Stage III (3)	32	2.64 (0.89)		28	3.21 (0.8)		20	3.22 (0.72)	
Other chronic disease (b)			0.712			0.425			0.619
No	96	2.73 (0.88)		82	3.12 (0.72)		60	3.16 (0.69)	
Yes	34	2.79 (0.82)		25	2.98 (0.71)		13	3.05 (0.71)	
BC subtype (d) †			0.269			0.216			0.298
Luminal B1 (1)	53	2.70 (0.83)		44	2.96 (0.73)		28	3.04 (0.72)	
Luminal B2 (2)	18	2.99 (0.70)		16	3.16 (0.74)		12	3.00 (0.84)	
Triple Negative (3)	32	2.58 (0.81)		23	3.03 (0.76)		15	3.12 (0.60)	
HER2 over expression (4)	27	2.87 (1.06)		24	3.33 (0.60)		18	3.41 (0.58)	

a: Pearson's correlation coefficient.

b: Independent *t*‐test.

c: Kruskal–Wallis test (post hoc test: Mann–Whitney *U* test with Bonferroni's correction).

d: One‐way analysis of variance (ANOVA) (Scheffe's post hoc test).

†: Variance unequal (robust ANOVA with Games–Howell method).

Abbreviations: BC, Breast cancer; HER2, human epidermal growth factor receptor 2.

T1: first chemotherapeutic cycle; T2: second chemotherapeutic cycle; T3: final chemotherapeutic cycle.

### 4.3 Analysis of changes in self‐efficacy

**FIGURE 1:**
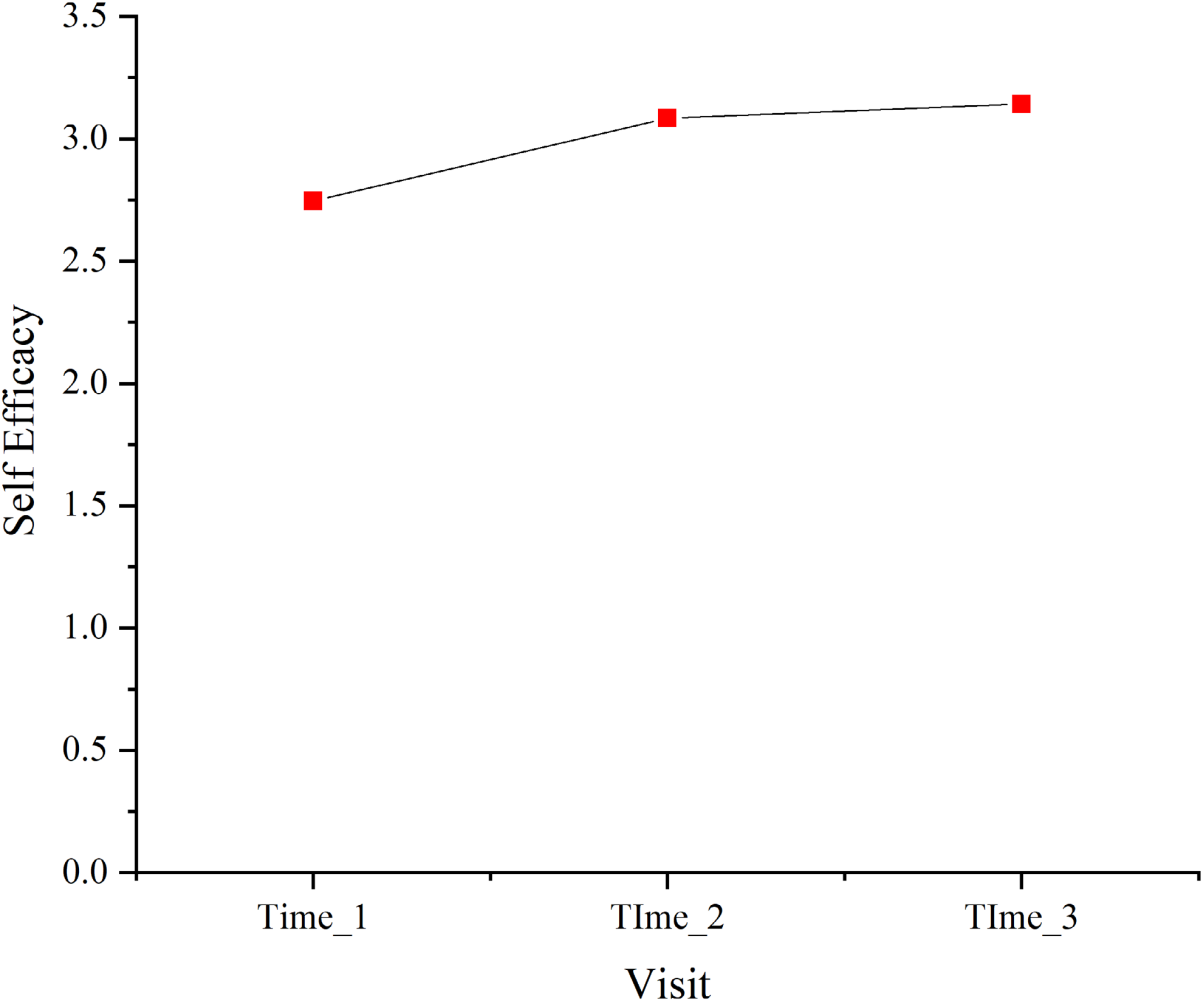
Predictive value of the repeated measures of self‐efficacy in the generalized linear model. Horizontal axis: before the first (Time_1), second (Time_2) and last (Time_3) cycles of adjuvant chemotherapy

**FIGURE 2:**
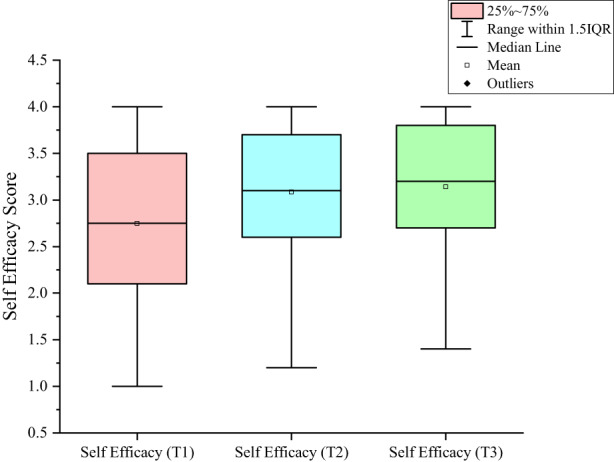
Self‐efficacy boxplots. Horizontal axis: before the first (Time_1), second (Time_2) and last (Time_3) cycles of adjuvant chemotherapy

In the GEE analysis, three repeated measurements of GSES were used as variables. The measurement time points (T1, T2 and T3) and the demographic variable of religious beliefs (which was found to be statistically significant in the correlation analysis) were used as independent variables. The changes in the self‐efficacy of patients with BC undergoing adjuvant chemotherapy at different time points were investigated. After controlling for differences in the religious belief variable, self‐efficacy at different measurement time points exhibited statistically significant differences (*p* < 0.001); the self‐efficacy scores at T2 and T3 were higher than those at T1 (regression coefficients of 0.341 and 0.382, respectively). The results of this analysis are presented in Table 4, Figure 1 and Figure 2. No significant difference was observed between measurements at T2 and T3 (*p* > 0.05).

**TABLE 4:** Generalized estimating equation analysis of repeated measures of self‐efficacy and demographic variables

Variable	*b*	SE	*p* value	95% CI	
				Lower	Upper
Time point					
T2 vs. T1	0.341	0.091	<0.001	0.163	0.519
T3 vs. T1	0.382	0.103	<0.001	0.180	0.585
Religious belief					
Yes vs. No	0.152	0.112	0.174	−0.067	0.370

*Note*: Dependent variable: self‐efficacy score.

Abbreviations: CI, confidence interval; SE, standard error.

## 5 DISCUSSION

This study showed that the self‐efficacy of patients with BC undergoing adjuvant chemotherapy for the first time was the lowest before the first chemotherapy cycle (T1), significantly increased before the second cycle (T2) and was maintained at this level until the third cycle (T3). Self‐efficacy before T2 was slightly (but not significantly) lower than that before T3.

A possible explanation for this result is that patients with BC undergo the first chemotherapy cycle within a short period after surgery and that they are often unsure of their ability to cope with the physical reactions and undesirable effects caused by chemotherapy during this period and are thus prone to high levels of uncertainty and anxiety (Zhang et al., 2015) and low self‐efficacy. However, after undergoing the first chemotherapy cycle, the procedures and undesirable effects of chemotherapy become clearer over time. As a result, the fear and sense of uncertainty regarding chemotherapy decreases, and patients gradually learn the appropriate self‐management behaviours that should be adopted; this in turn leads to increased self‐confidence and self‐efficacy (Foster et al., 2015).

This phenomenon also confirms the degree of self‐efficacy reported in the literature, including the reported effects of chemotherapy on quality of life, stress reduction, decision‐making and positive attitude (Brandão et al., 2017). Self‐efficacy has also been reported to be negatively correlated with anxiety and depression (Curtis et al., 2014). The fear, denial and the role of treatment‐related adverse effects in hindering daily activities can initially result in decreased self‐efficacy. However, among the three measurement time points, an obvious difference in self‐efficacy was only observed between before T1 and T2. This indicates that, after undergoing T1, the patient gradually gains clarity regarding the steps of chemotherapy and the undesirable effects, reducing their fear and uncertainty pertaining to treatment. This is consistent with a German questionnaire follow‐up study on the fear of treatment (including surgery, chemotherapy and radiotherapy) in 761 primary patients with BC, which showed that patients with BC feared chemotherapy more than surgery or radiotherapy and that this state of elevated fear continued until chemotherapy was complete (Singer et al., 2015). Our findings are also consistent with those of a study tracking 137 BC and colorectal cancer patients in the United States, showing that increased self‐efficacy is beneficial for positive attitudes towards improving mood and happiness in order to face the challenges caused by cancer (Papadopoulou et al., 2017).

This study also showed greater self‐efficacy at the second time point than at the third time point; however, this difference was not significant. This may have been attributed to the sample size, which was not sufficiently large for the effect to be evident. Notably, with respect to the self‐efficacy of the patient after surgical treatment, self‐management ability and self‐efficacy increased when the anxiety experienced by a patient regarding the second chemotherapy cycle was reduced. We believe that if actual clinical care proficiency and experience are lacking, role model behaviour, observation and learning can be a substitute for experience and inspire confidence in mimicking behaviour, thereby improving self‐efficacy.

### 5.1 Limitations and recommendations

Owing to time, human resources and funding constraints, this study only recruited patients with BC from a medical centre in northern Taiwan, and the small number of participants cannot represent the entire population. Therefore, the representativeness and inferential value of the study are insufficient, and the results must be interpreted conservatively. The internal reliability of the representativeness was consistent (Cronbach's *α* of 0.97) and was also consistent with that of previous studies (Cronbach's *α* of 0.92) (Foster et al., 2015), which may also be due to consistency within the breast cancer groups. In future studies, we recommend expanding the scope of inclusion and adopting a random sampling design to achieve higher inferential values. In addition to chemotherapeutic agents, the BC subtype is an important factor influencing the precise medical treatment of BC. However, this classification is not widely used in nursing care. We recommend that the BC subtype classification be employed for subdividing clinical applications for nursing care and health education and for identifying interventional measures that can improve patient self‐efficacy. This will help increase the positive stress‐response strategies of patients with BC and effectively reduce their anxiety and depression. In addition, cancer care is not only achieved at the individual level but also involves the concept of family care. During the interviews, the interviewees often mentioned family members, relatives and friends who were helpful to them, indicating that family members had a major influence on their lifestyle and ability to withstand stress. Therefore, we recommend adding family‐related variables, such as family cohesion and family support, to the research tools. Finally, although the present study adopted a long‐term follow‐up design, it also followed a structured questionnaire interview process, making it impossible to understand the quality of life of patients with BC during T1. Therefore, we recommend the inclusion of qualitative interviews in future studies to explain the results of this study in greater depth.

## 6 CONCLUSION

The present study used Bandura's self‐efficacy theoretical framework to improve the understanding of patients with BC in their ability to undergo and control expected events under different conditions, which may affect their attitudes, efforts, persistence and thinking of positive and negative pressures. The results showed that self‐efficacy affects the behaviour and self‐management of patients with BC facing the first chemotherapy cycle by reducing their expected fear and anxiety and providing them with an expectation of health outcomes. Adjustment of disease response strategies can also determine whether patients can persevere in their efforts when faced with difficulties and unpleasant experiences. Thus, medical personnel should guide patients with BC to face the disease with a more positive attitude and promote their self‐efficacy as an early interventional measure to improve disease control and quality of life.

## FUNDING INFORMATION

None.

## CONFLICT OF INTEREST

None.

## ETHICS STATEMENT

This study was approved by the Institutional Review of the National Taiwan University Hospital (No. 20170709RIN) for data collection between August 2017 and September 2018.

7

## Data Availability

The data that support the findings of this study are available on request from the corresponding author. The data are not publicly available due to privacy or ethical restrictions.
